# From Surveillance to Intervention: Overview and Baseline Findings for the Active City of Liverpool Active Schools and SportsLinx (A-CLASS) Project

**DOI:** 10.3390/ijerph15040582

**Published:** 2018-03-23

**Authors:** Nicola McWhannell, Lawrence Foweather, Lee E. F. Graves, Jayne L. Henaghan, Nicola D. Ridgers, Gareth Stratton

**Affiliations:** 1 Department of Sport and Exercise Sciences, University of Chester, Chester CH1 4BJ, UK; 2 Physical Activity Exchange, Research Institute for Sport and Exercise Science, Liverpool John Moores University, Liverpool L3 2AT, UK; L.Foweather@ljmu.ac.uk (L.F.); L.E.Graves@ljmu.ac.uk (L.E.F.G.); 3 Laude Lady Elizabeth Junior School, Entrada Norte de La Cumbre del Sol, Benitachell, 03726 Alicante, Spain; jaynehenaghan@googlemail.com; 4 Institute for Physical Activity and Nutrition (IPAN), School of Exercise and Nutrition Sciences, Deakin University, Geelong 3220, Australia; nicky.ridgers@deakin.edu.au; 5 Applied Sports Technology Exercise Medicine Research Centre, Swansea University, Swansea SA1 8EN, UK; G.Stratton@swansea.ac.uk

**Keywords:** intervention, obesity, fundamental movement skill, physical activity, fitness, cardiovascular, physical self-perception

## Abstract

This paper outlines the implementation of a programme of work that started with the development of a population-level children’s health, fitness and lifestyle study in 1996 (SportsLinx) leading to selected interventions one of which is described in detail: the Active City of Liverpool, Active Schools and SportsLinx (A-CLASS) Project. The A-CLASS Project aimed to quantify the effectiveness of structured and unstructured physical activity (PA) programmes on children’s PA, fitness, body composition, bone health, cardiac and vascular structures, fundamental movement skills, physical self-perception and self-esteem. The study was a four-arm parallel-group school-based cluster randomised controlled trial (clinical trials no. NCT02963805), and compared different exposure groups: a high intensity PA (HIPA) group, a fundamental movement skill (FMS) group, a PA signposting (PASS) group and a control group, in a two-schools-per-condition design. Baseline findings indicate that children’s fundamental movement skill competence levels are low-to-moderate, yet these skills are inversely associated with percentage body fat. Outcomes of this project will make an important contribution to the design and implementation of children’s PA promotion initiatives.

## 1. Introduction

The relationships between children’s physical activity (PA) and metabolic health are widely reported in the literature [1,2,3]. PA is independently associated with clustered cardiovascular disease (CVD) risk and is therefore a key component in preventing or reducing the onset of CVD in adulthood [4]. Health benefits are thought to increase with moderate and vigorous intensity aerobic-based activity and weight bearing activity to elicit bone health benefits [1,2,3,4].

### 1.1. Generating an Evidence Base: Sportslinx

SportsLinx was initiated through a collaborative agreement between Liverpool John Moores University and the local authority Sport and Recreation Department in 1995. The aim of this collaboration was to develop a project that would strengthen the relationship between primary schools, sport and recreation and Liverpool John Moores University. The core programme needed to meet the missions of partner organisations, and have the potential to inform partners about sport participation, fitness, dietary behaviour and lifestyles of children. The programme was based on the Northern Ireland Youth Fitness Project [5] and adapted to match the needs of participating stakeholders. The pilot SportsLinx programme included 1500 9–14 year old children from an opportunity sample of 10 primary and 10 high schools in Liverpool. The pilot demonstrated that a health-related collaborative project was feasible and schools’ compliance was good. Further conversations with teachers and children supported wider strategic implementation. The SportsLinx project was implemented in Autumn 1996 and involved around 2500 children per year until 2001 and 4500 children each year thereafter. The programme was implemented in a partnership between Liverpool John Moores University and the local authority Departments of Sport and Recreation and Public Health.

Details of the SportsLinx project including methods and protocols were first reported by Taylor et al. [6]. Subsequent publications by Stratton et al., Fairclough et al., and Boddy et al. [7,8,9] reported decreasing trends in fitness, and increasing trends in overweight and obesity and sedentary behaviours over a decade. Data also demonstrated that deprivation had little effect on outcomes whereas large sex differences were evident. Stratton et al. [7] further demonstrated that between 1996 and 2004 body mass index (BMI) increased in the least fit, average fit and most fit groups, while fitness had a significant downward trend. Fairclough et al. [10] also reported that children were highly sedentary. Boys spent more time playing sport, watching TV and playing video games than girls, whilst children from higher socioeconomic status (SES) groups spent more time playing sport than those in lower. Overweight girls were more likely to spend time on the internet than their normal weight peers. Further analysis of data found that BMI had plateaued in boys and decreased in girls, whereas cardiorespiratory fitness continued to decline across the whole population [9]. The Sportslinx data demonstrated the need for population-level interventions to promote PA, healthy eating and healthy lifestyles in 9–11 year-old children in Liverpool.

### 1.2. Designing Interventions

The SportsLinx research group noted the trends in the surveillance data in 1998 and started to plan interventions. As a result, a smaller number of exploratory studies that aimed to increase PA by changing school playgrounds were completed [11,12]. These subsequently led to a randomized controlled trial (RCT) that demonstrated that changing the school playground physical environment increased children’s PA [13]. In another intervention in physical education, we demonstrated that physical education teachers could intensify PA during lesson time [14]. The limitations of these studies were that they did not measure habitual PA; they did not quantify the effect of increasing the quantity of PA on the quality of movement such as motor competence, and they did not include an outcome variable driven by national policy.

Systematic reviews also support the development of robust interventions using rigorous measures. Mears and Jago [15] recently reviewed the effectiveness of after school PA interventions and found mixed results, with findings limited by the methodological quality of study design, and recommended that research designs were improved. Other reviews found that school-based interventions increased the proportion of children engaging in moderate-to-vigorous PA (MVPA) during the school day [16] and that programmes that ran for one school year were most effective [17]. Unlike the studies reviewed by Mears and Jago [15], these were not focused on after school provision. Further, interventions that have been implemented have not included robust measures of exposure (PA, structured exercise) or outcome (direct measures of body composition, motor competence, CVD risk factors, and objective measures of PA). Furthermore, only a small number of studies have been implemented in UK children [16,17,18], and more research is needed.

### 1.3. The Active City of Liverpool, Active Schools and SportsLinx (A-CLASS) Project

For over a century, physical education has been consistently used as a vehicle to promote PA, fitness and health [19]. Between 1997 and 2007, there was a significant investment in physical education curriculum time in schools in England. In 2002, the Physical Education, School Sport and Club Links (PESSCL) strategy was launched by the Labour government [20]. The objective of PESSCL was to increase the quality and quantity of physical education and sports opportunities for children aged 5–16 years old by providing two hours of high quality physical education per week. The two-hour aspiration was superseded in 2004 with the “four-hour offer” of high quality physical education and school sport (PESS) per week, both within and beyond the curriculum [21]. The “four-hour offer” entitled children to two hours of curriculum physical education and two hours of organized after-school sport.

The purpose of the A-CLASS Project was to measure the effect of this “four-hour offer” on children’s PA, health and motor competence. The A-CLASS design is therefore novel in that it is built on (i) a national policy and (ii) it examines the relative effectiveness of school-based interventions on both children’s health (i.e., obesity, cardiometabolic risk) and developmental outcomes (fundamental movement skills). Further, we sought to address some of the limitations in the literature [22] by using robust research tools and methodologies. We created a novel laboratory to community approach that aimed to quantify the effectiveness of structured and unstructured PA programmes on children’s PA, fitness, body composition, bone health, cardiac and vascular structures, fundamental movement skills, physical self-perception, and self-esteem [23].

The Medical Research Council (MRC) guidance on developing and evaluating complex interventions recommends a five-phase development approach based on theory and evidence of need [24]. The A-CLASS Project followed this approach. The preclinical and phase I were built on eight years of data from the SportsLinx project (*n* = 20,000). Subsequently phases II (exploratory trial) and III (RCT) were designed around the “four-hour offer” national policy for physical education and school sport (DfE 2004). The phase II exploratory trial proved that the study design and approach was feasible and effective [25,26,27], with promising evidence of effects on fundamental movement skills [25], cardiac function [27], and body composition [26]. As a result of the exploratory work, adjustments to the design were made prior to the study progressing to a full RCT.

The primary aim of this paper is to detail the decisions related to the design and methods for the A-CLASS Project trial. The intervention aimed to improve both developmental and risk-associated markers, specifically: fundamental movement skill competence, deemed an important marker of physical development [28], and a reduction in percentage body fat, as an important marker of disease risk [29]. A secondary aim of the paper is to present baseline descriptives and cross-sectional associations between the primary outcomes. To date, studies exploring relationships between body composition and skill competence have typically used BMI or waist circumference, with most reporting an inverse association [28,30]. However, the sensitivity and accuracy of such proxy measures of fatness is questionable, and therefore more research using reference methods of body composition such as dual energy X-ray absorptiometry (DXA) is warranted. Slotte et al. [31] recently found better skill proficiency was significantly and negatively associated with lower percentage body fat, as measured by DXA, in a sample of 304 eight-year-old Finnish children. However, no data exists for British children and further evidence is needed.

## 2. Materials and Methods

### 2.1. Study Design

The A-CLASS Project was a four-arm parallel-group school-based cluster randomised controlled trial, conducted within the metropolitan city of Liverpool (UK) between September 2006 and November 2009. The project received institutional research ethics committee approval (05138), and the trial is registered (ClinicalTrials.gov identifier NCT02963805).

**Figure 1:**
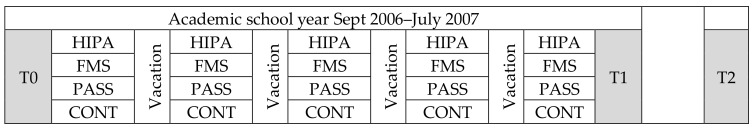
A-CLASS intervention design. Note: HIPA refers to High Intensity Physical Activity intervention; FMS refers to Fundamental Movement Skill intervention; PASS refers to Physical Activity Signposting Scheme intervention, CONT refers to the Control. T0 = baseline data collection (September 2006–mid-October 2006); T1 = 9 months post-intervention follow-up (June–mid-July 2007); T2 = 3 year follow-up (October 2009).

Data collection occurred over 36 months with three time points of baseline (month 0/T0, September to mid-October 2006), end of intervention (9 months/T1, June to mid-July 2007) and follow-up (3 years/T2, October 2009). At each time point participants attended University laboratories for individual assessments (anthropometry, bone and body composition, CVD risk factors, cardiorespiratory fitness), had their habitual PA objectively assessed using accelerometers, and were assessed for fundamental movement skills at their respective school. Prior to laboratory visits, participants were instructed to fast for a minimum of 8 h and avoid strenuous exercise for 24 h. The intervention ran over 9 months to replicate an academic school year. No intervention was delivered during school holiday/vacation periods. Figure 1 illustrates the intervention design, displaying time points for measurement and the intervention groups.

### 2.2. Participants and Settings

#### 2.2.1. Organisational Level

Government-funded primary schools were targeted by the research team in May–July 2006. The schools were assessed for eligibility based on their size (student enrolment >400 primary school (4–11 years old); >250 junior school (8–11 years old)), current afterschool club provision (limited), school sport facilities available for use (bi-weekly), and classified low socioeconomic status (SES). School postcode was used to determine low SES, which was defined as an Index of Multiple Deprivation less than 40 [32]. Sixteen schools met the inclusion criteria and signed consent was obtained from the head teachers for pupil recruitment, study contact, laboratory visits during school time and access to school facilities. Eight from the sixteen schools were randomly selected to take part using random number allocation. All schools had participated in the SportsLinx programme for over 7 years.

#### 2.2.2. Individual Level

All children in Year 5 (9–10 years old) in consenting schools received a verbal and written overview of the study through a researcher-led study information session held at the respective schools. Children provided written assent and a parent/guardian provided written consent to participate in the study. Medical questionnaires were distributed to all children who agreed to take part in the study. Self-reported stature and body mass were used in order to calculate BMI (kg/m^2^). BMI was used as guide to target the children who were overweight or at risk of being overweight according to UK age- and sex-specific cut-off thresholds [33]. Generally, 20–25 children with the highest BMI within each school, who were free from the presence of chronic disease, metabolic disorders, motor or coordination difficulties and prescribed medications including steroids inhaled by asthma sufferers were then enrolled to participate in the project. There was no racial or gender bias in the selection of participants.

### 2.3. Intervention

In a two-schools-per-condition design, using a computer generated procedure, participating schools were randomly allocated to one of four treatments to reduce risk of contamination effects across the trial. Treatment arms in this four-arm parallel-group cluster randomised controlled trial (RCT) included a high intensity PA (HIPA) intervention group, a fundamental movement skill (FMS) intervention group, a PA signposting (PASS) intervention group and a control group (usual practice). In a two-group-per-condition design, the equivalence of groups at baseline is not secure; to attempt to partially address this threat to validity, the schools selected were as similar as possible for the distribution of age, mass and deprivation. Each condition contained the children with highest BMI scores from each school; thus, the number of children varied slightly between intervention groups according to the number of children excluded for medical reasons, and to accommodate children with above average BMI in one school compared to another. The four-arm design was created to compare different exposures. The “Switch-Play” project [34] was used to guide the design of the PA signposting scheme (PASS) [35]. The high intensity group followed a programme where the instructor focus was on maintaining a high heart rate during multi-games activity, whereas the instructors of the FMS group led similar multi-games activities but focused their instruction on skill development as opposed to high levels of movement.

#### 2.3.1. Treatment Groups

1. High Intensity Physical Activity (HIPA)

The HIPA programme was based upon the STEX programme trialled in the feasibility study [26]. Children attended 60 min after-school club sessions twice a week for 26 weeks during school term time. After school sessions took placed in the sports-hall or outdoor space (depending on availability of facilities and weather conditions) at intervention schools. The activity sessions were delivered by trained multi-skills coaches with a variety of coaching qualifications and experience in coaching children. The sessions included a combination of high intensity activities (playground-style games and circuit training activities) which aimed to keep children moving and maintain a mean heart rate of above 70% of the age-predicted maximum heart rate (~145 beats/min) for the duration of the session to confirm the vigorous nature of the sessions. This was verified by intermittent heart rate (HR) monitoring (Polar Electro, Kempele, Finland). A-CLASS coaches delivered and monitored sessions and increased the intensity over time accordingly to allow for the children to progress. The mean HR for HIPA sessions was 150 beats/min, with children spending 52 min at this intensity during the session.

2. Fundamental Movement Skill (FMS)

The FMS programme was introduced into the intervention following the feasibility study [25] to assess the effect of skill based structured exercise. Children attended two 60 min after-school club sessions per week for 26 weeks during school term time. After school sessions took placed in the sports-hall or outdoor space (depending on availability of facilities and weather conditions) at intervention schools. The activity sessions were delivered by trained multi-skills coaches with a variety of coaching qualifications and experience in coaching. Each session focused on improving two FMS from the vertical jump, hop, sprint run, dodge, kick, catch, overarm throw, and strike. All skills were taught in equal quantities. The intervention programme was planned using activity resources designed by the Youth Sports [36]. Each session was designed to maximise participation and enjoyment, and consisted of a variety of games, drills and self-learning activities and offered numerous opportunities for practice. Skill components were taught to the children using simple learning cues, and skill related questions were used to develop purposeful feedback. The mean HR for FMS sessions was recorded at 141 beats/min, with children spending 55 min at this intensity during the session.

3. Physical Activity Signposting Scheme (PASS)

The PASS lifestyle intervention focused on increasing habitual PA and reducing time spent in sedentary behaviours. PASS was adapted after the initial pilot, which demonstrated poor compliance to the intervention [35]. In the current trial, a researcher visited the children once per week in 6 weekly blocks to set them an activity “mission” to take home and complete with family and friends. A total of twenty missions were set over 4 × 6 week blocks, each separated by a 6 week break. The intervention was designed based on the theoretical behaviour change model of the social cognitive theory [37] and was developed following action-research involving focus groups with children, parents and teachers. Additionally, parts of the intervention were derived from previous lifestyle interventions such as “Switch-Play” [38] and “Switch-Off, Get-Active” [39]. Each mission suggests a task as a prompt to participate in PA during the week and decrease sedentary activity [35]. The intervention aimed to encourage children to incorporate PA (no specific intensity) into their lifestyle using an intervention mapping approach. Children received a sticker on a wall chart for returning the mission; children were rewarded with prizes if all missions were returned in each block. On average, 58% of children returned all twenty missions. In addition to the “missions”, pedometers were given to the children as a promotional tool for the entirety of the project for the self-monitoring process of activity.

4. Control (CONT)

Children in the control group received British Heart Foundation leaflets that included information on heart health (given to all groups). Children participated in their usual school curriculum including two hours of physical education and school sport per week, both within and beyond the curriculum [20,21,40].

#### 2.3.2. Reward System

Within the HIPA and FMS intervention groups, children were awarded a reward point for every session attended; rewards (t-shirt, water bottle, pedometer, baseball cap, a Frisbee and yoyo) were given to children when a prerequisite number of points were achieved. Within the PASS intervention group, children received these rewards for completing and returning all missions in each 6-week period. Rewards were also given as incentives for PA monitoring compliance in the latter part of the study. The control group received rewards for attendance to baseline and post-intervention testing and for PA monitoring compliance. All children and participating schools received A-CLASS certificates to signify completion and participation in the project. The average attendance to a total of 70 sessions was 64% for HIPA and 68% for FMS after school interventions, whilst 75% of children adhered to PASS missions. Reasons for not attending sessions included absences from school, forgotten PE kit and school excursions.

### 2.4. Measures

#### 2.4.1. Anthropometry

A researcher trained to standards of the International Society for the Advancement of Kinanthropometry (ISAK) took all anthropometric measurements. Stature and sitting stature were both measured to the nearest 0.1 cm with a Leicester Height Measure (Seca Ltd., Birmingham, UK). Mass was assessed to the nearest 0.1 kg in light clothing without shoes using Seca scales (Seca Ltd., Birmingham, UK). BMI was calculated (kg/m^2^). Sitting height, mass, stature and age were entered into specific maturity offset calculations to predict years from peak height velocity, which is a somatic indicator of physical maturity [41]. Ethnicity was recorded from medical questionnaires completed by parents prior to commencement of study.

#### 2.4.2. Bone Mineral Content and Density

Bone mineral content (BMC; g) and areal density (BMD: g/cm^2^) of the total body (TB), femoral neck (FN) and lumbar spine (LS) were determined by means of dual-energy X-ray absorptiometry (DXA: Hologic QDR series Discovery A Fan-beam, Bedford, Massachusetts). The DXA was calibrated daily using the anthropomorphic spine provided by the manufacturer to assess accuracy of measurements. The coefficient of variation (CV) for repeated measurements of bone mineral by DXA analysis were BMCTB, 0.24%, BMDTB, 0.21%, BMCFN, 1.86%, BMDFN, 0.56%, BMCLS, 0.64% BMDLS, 0.40%. DXA provided both total body and segmental data on both bone and body composition and is considered the gold standard on bone densitometry [42]. The anterior–posterior lumbar spine L1–L4, femoral neck and total body scans were performed accordance with standard operating procedures and were performed in that order. With allowance for explanation of assessment procedures, the total time for DXA assessment was approximately 12 min for each participant. Participants were scanned in the supine position and wore lightweight clothing and no shoes. All scans were carried out by the same trained technician and were analysed after each assessment using Hologic QDR software for Windows version 11.2 (©1986–2001 Hologic Inc., Marlborough, MA, USA).

#### 2.4.3. Body Composition

Body composition was assessed by DXA and anthropometric skinfold thickness. The assessment of body composition using DXA has been validated against 4-compartment models in children [43]. The DXA provided absolute (kg) fat mass (FM) and lean tissue mass (LM) and relative (%) percent body fat (%BF) data and visceral fat from the total body scan. Distribution of body fat mass, lean mass and visceral fat was also achieved through segmental analysis. The calibration for body composition variables was performed weekly using a step phantom supplied by manufacturers. The coefficient of variation (CV) for repeated body composition measurements by DXA analysis were FM, 0.32%, %BF, 0.36%.

Multiple skin-fold measures were taken into account for morphological and fat distribution in addition to DXA data. This enabled the examination of relationships between skin-fold assessment and DXA assessment and other variables. Skinfold thickness was measured by eight skin-folds: triceps, subscapular, biceps, iliac crest, supraspinale, abdomen, front thigh and medial calf measured using Harpenden skin-fold callipers (Harpenden, UK). Girth measurements were taken using a tape measure (Lufkin W606PM, Apex Tool Group Ltd., Sparks, MD, USA) including waist circumference (natural waist), gluteal circumference (providing waist-to-hip ratio), arm girth relaxed, arm girth flexed and tensed, and calf girth. Bone breadths were also measured using a small sliding caliper (Rosscraft Innovations Inc., Vancouver, Canada) on the biepicondyles of the humerus and femur. All skin-fold and girth measurements were taken twice and repeated if measurement error was >5% for skinfolds, >1% for girths and >1% for bone breadths between first and second measurement. All measurements were taken according to the procedures outlined by ISAK [44]. The sum of 4 (triceps, subscapular, supraspinale, medical calf), 7 (triceps, subscapular, biceps, supraspinale, abdomen, front thigh and medial calf) and 8 (triceps, subscapular, biceps, iliac crest, supraspinale, abdomen, front thigh and medial calf) skin-folds was recorded to assess participants’ body fat.

#### 2.4.4. Fundamental Movement Skills

The eight skills assessed included the vertical jump, sprint run, hop and dodge (locomotor skills) and the kick, catch, overarm throw and strike (object-control skills). The skills were selected as they were considered suitable for the participant age range and varied enough to eliminate sex bias [45]. Additionally, the test battery represented skills that were perceived as important to successful participation in mainstream team sports such as football, netball, cricket and basketball. The skills were assessed using process-orientated measures, which focus on the way the skill is performed. Process-based measures were used to identify technical proficiency and thus offer useful guidance on the skill components that should be targeted during the intervention programme [46]. Assessment procedures involved using video analysis to check each performance of the skills against a checklist of components specific to each skill. The checklists were derived from the Australian resource: “Get Skilled, Get Active” [47], which detailed assessment procedures for 15 skills. The catch, overarm throw, kick, forehand strike, sprint run, leap, dodge and vertical jump tests were validated in a related manual [48], with acceptable alpha reliability coefficients (α = 0.70 or greater). This assessment tool was used for four reasons: Firstly, it was the only validated process-orientated measure for use in both children and adolescents. Second, results would enable comparisons to be made with published research at that time [38,49,50]. Third, checklists that list key descriptive components for each skill are useful for consistent scoring [51], and fourth the resource represented a functional test battery that could be adopted and implemented by primary educational professionals in future.

The task and skill component criteria for the assessment of each of the eight skills are shown in Table 1. Tests were conducted either in the school playground (weather permitting) or school gymnasium by one researcher using the same equipment at each site. Children were given a verbal description and single demonstration of the skill. Children were told to run, dodge and hop as fast as they could; to jump as high as they could; to catch as many balls as possible; and to throw, kick, and strike the ball with force rather than accuracy. Children performed each skill five times (except the sprint run and dodge, which was performed three times). The order of skill assessment was standardised throughout. Recordings of all participants were taken from identical angles and distances, with the video camera placed on a tripod during the testing. The data was then converted to DVD format for analysis to enable the assessor more precision when analysing skills performed high speed using slow-motion playback. During analysis, the skill component was recorded as present (scored ‘1’) if it was observed in four out of five trials (two out of three for sprint run and dodge). The number of skill components checked as present in each of the 8 skills was summed to create an overall skill score (max score 48) for use in the analysis. A single trained assessor conducted all fundamental movement skill assessments. Before data collection, an observer reliability study was conducted in 20 children (10 boys, 10 girls) randomly selected from the sample. Intra-rater reliability was established using a one-week test–retest study with kappa of 0.87 (90% CI: 0.83 to 0.91). Although only one assessor completed the analysis in this study, inter-rater reliability between this assessor and another trained observer was checked on pairs of 96 scores and calculated with kappa as 0.77 (90% CI: 0.71 to 0.83).

#### 2.4.5. “Pre-Clinical” Cardiovascular Disease Risk Factors

Brachial artery systolic and diastolic blood pressures and heart rate (Bosch and Sohn, Zollernalbkreis, Germany) were assessed after 5 min of lying in the supine position. Three measures were taken, with the lowest value noted.

Echocardiography was used to produce 2D image slices of the heart resulting in real time structural images of the myocardium. A trained ultrasound technician performed all ultrasound measurements. Two-dimensional/B-mode, M-mode, Doppler and tissue Doppler imaging (TDI) echocardiographic scans were performed using a portable ultrasound system (Mylab30CV system, ESAOTE, Firenze, Italy). All system settings including gain, filter, PRF, sector size and depth were adjusted to optimise the image quality.

Cardiac measurements included assessment of the left ventricular (LV) structures and LV mass [52,53,54,55]. With the participant in the left lateral decubitas position and following 10 min of quiet rest, left ventricular structures and LV mass were assessed using parasternal long axis images and M-mode scans at the level of the mitral valve with a 2.5 MHz phased array transducer. End-diastolic and end-systolic wall thickness and cavity dimensions were measured according to the American Society of Echocardiography [53,54,55]. Left ventricular mass was calculated according to a validated, regression-corrected formula [52]. LV mass was divided by height^2.7^ (included children) [53] to provide a size independent index of LV mass.

From the apical four-chamber view, Doppler recordings were taken of mitral inflow by placing a 2 mm sample volume at the tips of the mitral leaflets and parallel with flow. Peak early (E) and late/atrial (A) flow velocities were obtained and E/A ratio reported. In the same view, pulsed tissue Doppler (TDI) velocities were obtained from the septum at the mitral annulus using a 2 mm sample volume. Peak early diastolic (E’) and late diastolic (A’) myocardial tissue velocities were recorded and E’/A’ ratio was derived.

Carotid intima-media thickness (IMT) was assessed with a 10–15 MHz linear transducer on both the left and right sides of the neck. In the supine position, participants positioned their head away from the side of interest with their neck slightly extended. Ten millimetre segments of the far wall of the common carotid artery (CCA) 1–2 cm proximal to the carotid bulb were imaged. Four images were taken bilaterally and analysed off-line (IMT.LAB version 1.1, Pie Medical Equipment, Maastricht, The Netherlands). This software provided average mean and maximum values for carotid IMT via tracking the interfaces of the lumen-intima and media adventitia.

For a cohort of *n* = 30, scans were taken three times and analysed separately by the researcher and blinded to previous scores. From this, repeated measures ANOVA and ICC for systematic and random error were calculated. These were as follows: LVM f = 1.895 (*p* = 0.159), ICC = 0.862 (*p* = 0.000); E/A f = 0.710 (*p* = 0.496), ICC = 0.731 (*p* = 0.000); TDI f = 0.016 (*p* = 0.984), ICC = 0.794 (*p* = 0.000); and cIMT f = 2.323 (*p* = 0.107), ICC = 0.555 (*p* = 0.000).

#### 2.4.6. Cardiorespiratory Fitness

Peak oxygen uptake (.VO_2peak_) was assessed on a treadmill (H P Cosmos, Traunstein, Germany) during a discontinuous incremental protocol to volitional exhaustion, adapted from Armstrong [56]. All tests were conducted by the same trained technician, in physiologically neutral conditions. Children were habituated to laboratory conditions and underwent a familiarization period of walking and running on the treadmill prior to the test. The protocol consisted of 3-min stages, with a 30 s rest interval between each stage. The test started at an initial velocity of 4 km·h^−1^, which was increased by 2 km·h^−1^ for each subsequent stage. Treadmill gradient remained at 1.0% throughout the test [57]. The test was terminated at the point of volitional exhaustion when the child was unable to continue despite strong verbal encouragement.

A paediatric facemask (Hans Rudolph, Kansas City, MO, USA) covering the nose and mouth was secured via an adjustable nylon harness prior to test commencement. During the test oxygen uptake (VO_2_) and carbon dioxide production (VCO_2_) were measured breath-by-breath with an online system (Jaeger Oxycon Pro, Viasys Health Care, Warwick, UK). On all test days, vanes were calibrated using known volumes of flow rate (0.2 and 2.0 L/s^−1^) and the gas analysers against known concentrations of gases (0.5% CO_2_ and 20.5% O_2_). Respiratory variables were averaged over 15 s epochs. HR was monitored continuously (Polar, Kempele, Finland) and all participants wore a uni-axial accelerometer (GT1M model, Actigraph, Pensacola, FL, USA), mounted on the right hip, which recorded data every 5 s.

VO_2Peak_ was determined as the highest 15 s VO_2_ value at steady state (between 120 s and 180 s of each stage). VO_2_ was accepted as a maximal index when participants exhibited any of the following subjective indicators of maximal effort: unsteady gait, hyperpnea, facial flushing, sweating. These were confirmed by at least one of the following criteria also being satisfied: a respiratory exchange ratio (RER) ≥ 1.00, or HR ≥ 195 beats·min^−1^ [56].

#### 2.4.7. Physical Activity

Habitual PA was measured during waking hours over a period of seven consecutive days using a uni-axial accelerometer, which records movement in the vertical plane (GT1M model, ActiGraph, Pensacola, FL, USA). The ActiGraph has been shown to be a reliable and valid measure of PA in children and correlates reasonably well with doubly-labelled water derived energy expenditure [58,59,60,61]. In order to capture the full nature of children’s PA, which is characterised by transient, intermittent short bursts of high intensity activity ([62], the accelerometer was programmed to record PA data every 5 s. Children were asked to wear the monitor on the right hip using a tightly fitted elastic belt, during all waking hours except when swimming or bathing. All data were downloaded using ActiLife software (version 2.1.8; ActiGraph LLC, Pensacola, FL, USA). The total volume of PA including all intensities was termed the total PA and measured in counts per minute (cpm). For inclusion in the analyses, children were required to have produced counts for 9 h a day (between 6 a.m. and midnight) for at least three days. Three days of PA measurement have demonstrated an acceptable reliability coefficient of 0.7 [63]. Evenson cut-points were used to estimate time spent in sedentary, light, moderate and vigorous PA [64]. These cut-points have been recommended for use in children [65].

#### 2.4.8. Physical Self-Perceptions and Self-Esteem

Physical self-perceptions were assessed using the Children and Youth Physical Self-Perception Profile (CY-PSPP) [66], which was validated for use in young children [67] and was reported to have good stability and internal consistency across subscales (Cronbach alpha = 0.79 to 0.92) [66]. The questionnaire comprised of 36 questions, representing six scales (6 items per scales): global self-esteem, the domain of physical self-worth, and subdomains sports competence, physical strength, body attractiveness and physical condition. Each question consisted of two statements and used a structured alternative format. Each child read two statements for each item, and decided which “child” they were most like, and marked (X) if it was “really true” or “sort of true” for them. Children completed questionnaires independently during visits to the university laboratory. A researcher verbally described the questionnaire slowly and carefully to children to eliminate confusion, and was available to offer further assistance where necessary. Every question is scored from one to four (1 meaning low self-perception, 4 meaning high self-perception). Scores for each sub-domain were summed (range 6–24).

Eight further items were added to determine perceived competence in each of the assessed fundamental movement skills. This was done in order to include lower order facets/sub-facets of perceptions of competence, which matched fundamental movement skill competence tasks. The additional items were based on those included by Southall et al. [68] and were written in a similar format to the other CY-PSPP questions. The perceived skill competence items were summed to create composite perceived total, locomotor and object-control skill competence scores. 

### 2.5. Sample Size and Statistical Power

It was feasible to recruit eight schools and randomly assign two schools to each of the four arms. With an estimated number of consenting Year 5 pupils of 20 per school, the original planned sample size was around 40 participants per arm. Allowing for 10% attrition at 9 months (T1) and a design effect of 1.2 to account for school-level clustering (ICC of 0.01), our effective sample size was expected to be around 30 participants per arm. With an anticipated attrition of up to 50% for the 3-year follow-up time point (T2), our expected effective sample size was only 15 participants per arm. Group comparisons at this time point were therefore defined as exploratory. The actual power at 2P = 0.05 (not observed power) afforded by the trial to detect targeted effect sizes of 3 units for total FMS skills score and 2 percentage points for body fat % will be calculated from the trial data, alongside, if appropriate, the exaggeration ratio (the degree to which the magnitude of the true effect might be overestimated, expressed as a factor).

### 2.6. Statistical Analysis

#### 2.6.1. Baseline Descriptives and Cross-Sectional Analyses

To analyse fundamental movement skills, the number of components of each skill correctly demonstrated by each child was summed to give a score for each skill. A total skill score was created by summing the total number of skill components checked as present in each of the eight skills assessed (the index could hypothetically range from 0 to 48). Two further composite scores were created: locomotor skills (sum of components successfully demonstrated in the hop, vertical jump, sprint run and dodge; range 0 to 24) and object-control skills (sum of components successfully demonstrated in the catch, throw, strike and kick; range 0 to 24). Additionally, each skill was scored by creating a binary outcome variable of “proficient” (scored 1) versus “non-proficient” (scored 0), with “proficiency” defined as demonstration of all, or all but one, of the listed skill components. Failure to achieve this standard was classed as “non-proficient”.

All analyses were completed with IBM SPSS Statistics for Windows, version 23 (IBM SPSS, Tokyo, Japan). Descriptive statistics were undertaken for all continuous variables (mean, standard deviation). Frequency statistics were used to report the proportion of participants achieving proficiency for each skill and classified as non-overweight or overweight, in accordance with International Obesity Task Force cut-offs [69]. Independent t-tests examined sex differences for continuous variables, whilst Chi-square tests were used to assess differences in prevalence of skill proficiency. Univariate analysis of covariance (ANCOVA) were used to calculate sex differences on total skill score, locomotor and object-control skill composite scores, controlling for any differences on variables (maturity offset, percentage body fat, ethnicity, deprivation) which may confound, moderate, or mediate relationships. Forced entry linear regression analyses examined whether fundamental movement skill composite scores predicted percentage body fat, whilst controlling for sex, maturity offset, ethnicity, and deprivation. Squared semi-partial correlations were calculated to indicate the unique variance attributed to each predictor.

#### 2.6.2. Cluster Randomized Controlled Trial

There are too few clusters per arm to account robustly for the hierarchical data structure using linear mixed (multilevel) modelling, generalised estimating equations, or clustered robust standard errors. Therefore, data were analysed at the individual level, with standard errors inflated by the square root of the design effect. The change in FMS score (or body fat %) from baseline to post-intervention (9-months) was compared between arms using a regression model (ANCOVA), adjusting for baseline FMS score (or baseline body fat %), sex, maturity offset, ethnicity, and SES deprivation. For each primary outcome, there were three planned comparisons comprising each of the 3 interventions vs. usual practice. Point estimates were derived together with uncertainty expressed as 90% confidence intervals. For the primary outcomes, a purely exploratory sub-group analysis was conducted using a sex-by-intervention group interaction term to examine the potential for differential intervention effects in boys vs. girls. Secondary outcomes were analysed using the same general modelling approach, but with no inferential emphasis placed on the results. Where appropriate, a principled method was applied to address missing data (e.g., multiple imputation or full information maximum likelihood). Analysis was conducted according to the intention-to-treat principle (as randomised).

## 3. Results

### 3.1. School and Participant Study Flow

**Figure 2:**
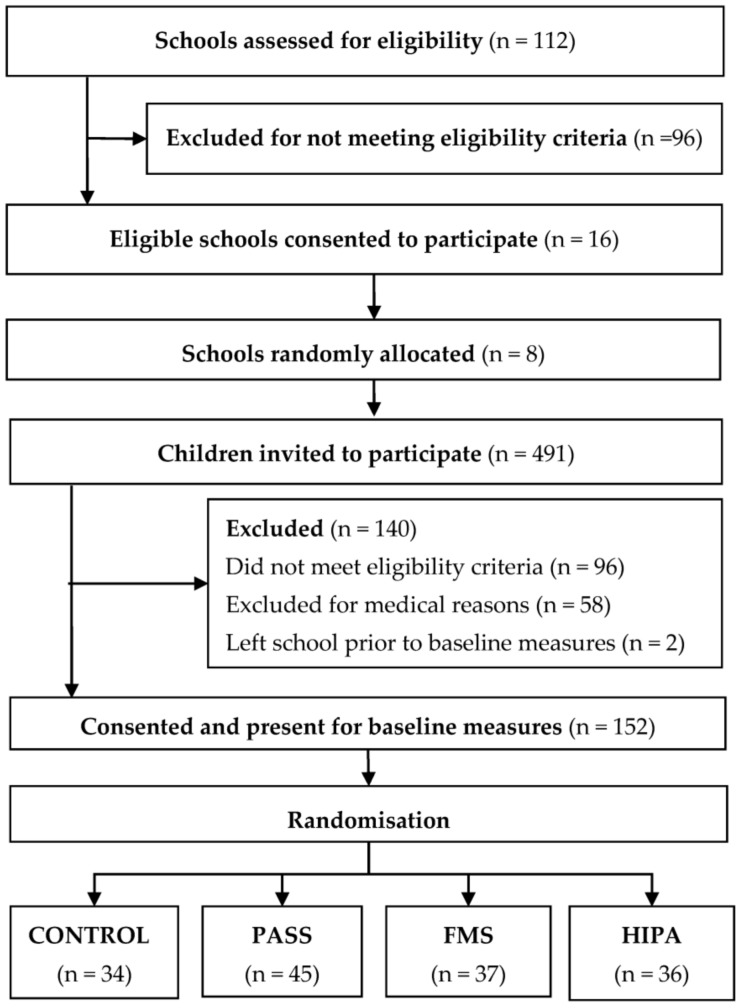
School and participant flow to baseline assessments. Note: HIPA, High Intensity Physical Activity intervention; FMS, Fundamental Movement Skill intervention; PASS, Physical Activity Signposting Scheme intervention.

Figure 2 shows school and participant flow through to baseline assessments. In total, 491 children were assessed for eligibility in the study. Informed consent was received from 292 children (59% response rate), though 140 children (48%) were excluded for not meeting the inclusion criteria or for medical reasons, or because they either left school or were withdrawn by their school prior to baseline measurements. The baseline sample therefore included 152 children (90 girls, 62 boys).

### 3.2. Baseline Characteristics

Of the 152 children (62 boys, 90 girls) in the baseline sample, 146 children (39% boys) completed measurements for both primary outcomes (i.e., body fat percentage and fundamental movement skills) and were included in the cross-sectional analyses. Missing data included three children who were absent on testing days, whilst another three children were not scanned by DXA following requests from parents.

Descriptive statistics for the 146 children included in the cross-sectional analyses are shown in Table 2. Around three quarters of children lived in areas of high deprivation [32], with 38% of girls and 38% of boys classified as overweight or obese [69]. Compared to boys, girls were significantly closer to peak height velocity (maturity offset) and had higher percentage body fat. No other anthropometric sex differences were found.

Fundamental movement skill data are in Table 2. Boys demonstrated significantly more skill components than girls in the dodge, kick, catch, throw and strike. Boys scored highest in the four object control skills, which were girls’ lowest scoring skills. Girls performed best at locomotor skills including the hop, vertical jump and sprint run, with the latter two skills amongst boys’ lowest scores. The dodge was the worst skill for both boys and girls. Significant sex differences, favoring boys, were also observed for total skill score, locomotor skills and object-control skills composite scores. However, following adjustments for age, ethnicity, deprivation, maturity offset and percentage body fat, boys only scored significantly higher in total skill score (Adjusted mean difference 7.6 units, 95% CI: 4.5 to 10.7; *p* < 0.01) and object-control skills (Adjusted mean difference 7.2 units, 95% CI: 5.1 to 9.2; *p* < 0.01). There was no significant difference in locomotor skills (Adjusted mean difference 0.5 units, 95% CI: −1.2 to 2.1; *p* = 0.595).

**Figure 3:**
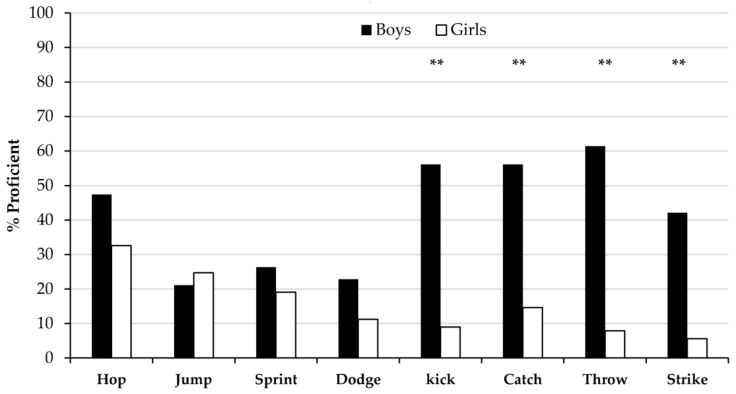
Prevalence of proficiency at fundamental movement skills (** *p* < 0.01).

Figure 3 shows the prevalence of proficiency (possessing all, or all but one, required components of a skill) at FMS for boys and girls. Overall, boys were significantly more proficient than girls (42% vs. 16%). Boys were more advanced than girls in seven out of the eight skills; however these differences were only significant (*p* < 0.01) in the four object control skills: the catch (χ^2^ = 25.3), overarm throw (χ^2^ = 44.1), strike (χ^2^ = 28.4) and kick (χ^2^ = 37.4).

Prevalence of proficiency amongst girls was very low. Only one-third of girls were rated as proficient in the hop, yet this represented their highest rated skill. The next best assessed skill was the vertical jump, with just 25% proficiency. Girls performed worst at the overarm throw, strike and kick, with over 9 out of 10 girls being classified as poor. Prevalence of proficiency was higher in boys. Over half of boys were classed as proficient in the overarm throw, kick, and catch. However, boys performed worst in the vertical jump, sprint and dodge, with over 75% non-proficient.

### 3.3. Main Analyses

Results of the standard multiple regression examining skill composite scores as a predictor of percentage body fat are in Table 3. Controlling for sex, maturity offset, ethnicity and deprivation, total skill score significantly predicted 5.2% of unique variance in percent body fat. Specifically, a one-unit increase in total skill score is associated with a 0.27% decrease in body fat (95% CI: −0.43 to −0.12). When the total skill score variable was removed from the regression model and replaced with the composite variables of locomotor skills and object-control skills (Model 2), locomotor skills significantly predicted 15% of unique variance in percent body fat. Specifically, a one-unit increase in locomotor skills score was associated with a 0.88% decrease in total body fat (95% CI: −1.14 to −0.61). Object-control skills weakly predicted two percent of unique variance in percent body fat but in the opposite direction: a one-unit increase in object-control score was associated with a 0.26% increase in percent body fat.

## 4. Discussion

This paper aimed to provide the background, study design and methods of an innovative programme of research that will make an important contribution to the implementation and design of children’s PA promotion initiatives and policy—The A-CLASS Project. A secondary aim of the paper was to present preliminary findings for participating A-CLASS children with respect to primary outcomes for positive development and risk (i.e., fundamental movement skills and percentage body fat, respectively), and to explore their cross-sectional associations. The fundamental movement skill results indicate that the prevalence of skill proficiency among this population was low-to-moderate, with boys more skillful than girls in object-control skills, but not locomotor skills. Further, skill competence was a significant predictor of percent body fat, suggesting that developing fundamental movement skill competence may be important for addressing childhood obesity.

In boys, prevalence of skill proficiency was low-to-moderate (ranging from 21% to 61%) and did not exceed 60% in any skill except the overarm throw. In girls prevalence of skill proficiency in girls was low (ranging from 6% to 33%), and only the hop had over 30% rated as proficient, whilst 9 out of 10 girls were rated as non-proficient in the throw, strike and kick. Skill proficiency may be low as children were recruited into the project from areas of high deprivation, with children from higher socioeconomic backgrounds more likely to possess higher levels of skill proficiency [70]. Children from more affluent areas may have greater access to facilities and be more likely to own sports equipment and therefore have increased opportunities to develop these skills. Nonetheless, the large proportion and pattern of primary school children rated as non-proficient in this sample is similar to results from more heterogeneous samples of similar-aged children in the United Kingdom [71] and Australia [50,72,73], which assessed skills using a similar assessment tool. The results suggest that there is significant potential to improve fundamental movement skill competence.

The low prevalence of skill proficiency is worrying given that children should master most fundamentals by eight years of age [51,74], and because evidence suggests that failure to master such skills may provide a barrier to participation in PA [28,30,75,76,77]. The development of fundamental movement skills is not automatic; in order to develop, skills need to be taught and practiced. Instruction time necessary to develop skill proficiency can range from 240–600 min, depending on the skill and developmental capability of the child [47]. Nevertheless, development of competence at these skills is influenced by a wide range of bio-psycho-social and environmental factors [78]. It may be that children in this sample were not sufficiently active and so lacked the opportunities to develop these skills, or that the PA experiences that children engaged in may have lacked the quality to facilitate improvements in such skills. For example, children may not have received the quantity or quality of coaching within physical education or sport necessary to reach proficiency in these skills. The training provided to primary school teachers in physical education has significantly declined [79] and many generalist teachers lack the necessary skills and confidence to deliver physical education [80,81]. School priorities are literacy and numeracy, with limited attention given to the provision of quality early learning experiences in physical education. While more evidence is needed on how best to train teachers to enhance fundamental movement skill development [82], the pressure on curricular time supports the need for after-school programmes to provide an additional opportunity for enhancing such skills [25].

The skills selected for assessment were considered age-appropriate and free from gender bias. However, overall, boys possessed more skill components and were more proficient than girls. Similar to previous studies which used similar methods [50,71,72,73], when adjusted for covariates, gender differences were significant in object control skills (boys better), but not locomotor skills. The observed gender differences in object-control skills may reflect the different sports and activities in which children typically participate in at this age. More boys play football, basketball and cricket than girls and so have further opportunities to develop and practice object control skills such as kicking, catching and throwing. Conversely, girls are more likely to participate in activities such as dance or gymnastics, which do not reinforce object-control skills. Differences are also formulated in unstructured settings such as school playtime. Boys are significantly more likely to participate in ball games and use equipment whereas girls prefer social and sedentary play, skipping or hopscotch [83,84]. This explanation indicates that gender differences are environmentally and culturally induced, rather than biological. If similar opportunities for instruction, practice, encouragement, and feedback are provided to both boys and girls then observed sex differences can be reduced.

Overweight and obese children suffer from bullying, name-calling and teasing, and may seek to avoid overt victimisation by withdrawing from PA [85,86], which, in turn, may limit their opportunities to develop skill competence. The results did reveal weak-to-moderate negative associations between fundamental movement skill competence and percent body fat. Specifically, after adjustments for confounders, total skill score significantly predicted 5.2% of the variance in percent total body fat. However, when skills were partitioned into locomotor skills and object-control skills, only locomotor skills were inversely associated with percent body fat, predicting 15% of variance, signifying a moderate association. This finding of a unique influence for locomotor skills is supported by a recent study in a UK primary school, which found that children with higher levels of locomotor skill proficiency had lower BMI and body fat percentage, as measured by skinfolds [87], though no such differences were observed for object-control skills. Furthermore, Southall et al. [68] observed that compared to their non-overweight peers, overweight children, as classified by BMI, had lower competence in locomotor skills but no difference was found for object-control skills. Moreover, Okely et al. [88] reported that non-overweight boys and girls were two to three times more likely to possess more advanced locomotor skills than overweight boys and girls, though object-control skills were virtually unrelated to body composition. Slotte et al. [31] did report that better object-control proficiency was associated with lower body fat percentage in Finnish children, as measured by DXA. However, the study did not adjust for confounders relating to both obesity and movement skills, which may have influenced the relationships causal pathway.

Locomotor skills may be more strongly associated with adiposity than object-control skills for several reasons. Firstly, object-control skills require less movement of body mass from one place to another than locomotor skills, and so may be less difficult to perform for overfat children [89]. Secondly, obese children can demonstrate abnormal gait patterns [90], which increases the energy cost of locomotion and may cause early fatigue, thus limiting time spent practising locomotor skills. Thirdly, abnormal gait and resultant biomechanical deficiency can cause musculoskeletal pain in knee and hip joints, again limiting participation [91]. Taken together, the results suggest that improving locomotor skills may be a plausible intervention strategy to reduce adiposity in children; however, experimental research is needed to determine the causal direction of this relationship.

The strengths of this study using baseline data from the A-CLASS Project are the use of DXA to assess percentage body fat, and the use of video analysis and a process-oriented, sensitive assessment of fundamental movement skills. However, due to the cross-sectional design, causality cannot be established, whilst the relatively homogenous sample may limit the generalisability of the findings. Nevertheless, outcomes from the cluster randomized controlled trial can be used to inform interpretations of cause and effect.

In summary, the preliminary baseline findings of the A-CLASS Project corroborate those from other studies examining fundamental movement skills in children. Prevalence of proficiency in skills was low-to-moderate, suggesting that there is great potential to improve such skills. It is especially important that children develop movement skills as competence was inversely related to percentage body fat. These findings support the need for developmentally-appropriate school and community programmes. Outcomes from The A-CLASS project will provide a comprehensive and robust examination of the effectiveness of three feasible school-based PA programmes on the PA, body composition, bone health, cardiac and vascular structures, fundamental movement skills and physical self-esteem of children.

## Figures and Tables

**Table 1:** Fundamental movement skill test battery and assessment criteria.

Skill	Task	Criteria
**Hop**	Hop as fast as you can over a distance of 15 m	1. Support leg is bent in preparation and then straightens to push off 2. Takes off and lands on forefoot 3. Swing leg moves in rhythm with support leg 4. Able to hop on both right and left legs 5. Head and trunk stable with eyes focused forward 6. Arms bent and move to assist leg action
**Vertical Jump**	Jump and touch the wall as high as you can	1. Eyes focused forwards or upwards throughout the jump 2. Crouch with knees bent and arms behind the body 3. Forceful upward thrust of arms as legs straighten to take off 4. Legs straighten in the air 5. Contact ground with front part of feet and bend knees to absorb force of landing 6. Balanced landing with no more than one step in any direction
**Dodge**	Dodge through a series of cones placed in zig-zag formation, 3 m apart	1. Bend knees during change of direction 2. Push off on outside of foot when changing direction 3. Body lowered during change of direction 4. Eyes focused in direction of travel 5. Can dodge to either side 6. Arms move to assist action
**Sprint run**	Run a distance of 30 m as fast as possible	1. Lands on balls of feet 2. Eyes focused forward, head and trunk stable throughout the run 3. High knee lift (thigh almost parallel to the ground) 4. Knees bend at right angles during the recovery phase 5. Arms bent at least 90 degrees 6. Arms driving forward and back in opposition to legs
**Kick**	Kick a size 4 football towards a target as hard as possible	1. Eyes are focussed on the ball throughout the kick 2. Forward and sideward swing of arm opposite kicking leg 3. Step forward with non-kicking foot placed near the ball 4. Hip extension and knee flexion of at least 90 degrees during preliminary kicking movement 5. Contact the ball with the top of the foot (a “shoelace” or instep kick) 6. Kicking leg follows through high towards the target after ball contact
**Catch**	Catch a tennis ball thrown underarm between 2–3 m high, and from a distance of 10 m	1. Eyes are focused on the ball throughout the catch 2. Feet move to put body in line with object 3. Hands move to meet ball 4. Hands and fingers positioned correctly to catch the ball 5. Catch and control the ball with hands only (well-timed closure) 6. Elbows bend to absorb force of the ball
**Overarm Throw**	Throw a tennis ball overarm as far as possible	1. Eyes are focused on the target throughout the throw 2. Stand side-on to the target 3. Arm moves in a down-ward and backward arc 4. Step towards the target with foot opposite throwing arm during the throw 5. Hip then shoulders rotate forward 6. Throwing arm follows through down and across the body
**Strike**	Using a t-ball stand and a foam baseball bat, hit a tennis ball as far as possible	1. Stand side-on to target 2. GRIP: hands next to each other, hand closest to handle end matches front foot 3. Front foot steps forward (weight transfers from back to front) 4. Hips then shoulders rotate forwards 5. Ball contact made on front foot with straight arms 6. Follow through with bat around body

**Table 2:** Descriptive statistics for children.

Characteristics	Total (*n* = 146)	Boys (*n* = 57)	Girls (*n* = 89)	*p*-Value
***Anthropometry***				
Age (years)	9.6 (0.3)	9.6 (0.3)	9.6 (0.3)	0.913
Maturity offset (years)	−2.4 (0.8)	−3.3 (0.5)	−1.9 (0.5)	0.000 **
Stature (cm)	138.5 (6.4)	138.9 (6.5)	138.3 (6.3)	0.556
Mass (kg)	36.7 (8.4)	37.0 (8.9)	36.5 (8.1)	0.737
BMI (kg/m^2^)	18.9 (3.2)	18.9 (3.4)	18.9 (3.1)	0.997
Body fat (%)	27.6 (6.5)	24.9 (6.9)	29.3 (5.7)	0.000 **
***Fundamental movement skills***				
Hop	4.0 (1.2)	4.2 (1.2)	3.9 (1.2)	0.126
Vertical jump	4.0 (0.8)	4.1 (0.8)	4.0 (0.8)	0.666
Sprint run	3.5 (1.1)	3.5 (1.2)	3.5 (1.0)	0.934
Dodge	2.8 (1.4)	3.3 (1.4)	2.5 (1.4)	0.001 **
Kick	3.5 (1.5)	4.5 (1.4)	2.9 (1.1)	0.000 **
Catch	3.4 (1.7)	4.5 (1.3)	2.7 (1.6)	0.000 **
Throw	3.3 (1.7)	4.7 (1.2)	2.4 (1.3)	0.000 **
Strike	3.4 (1.5)	4.4 (1.2)	2.9 (1.2)	0.000 **
Locomotor skills	14.4 (3.3)	15.1 (3.4)	13.9 (3.1)	0.028 *
Object control skills	13.6 (5.1)	18.2 (3.7)	10.7 (3.4)	0.000 **
Total skill score	28.0 (7.1)	33.3 (5.9)	24.6 (5.5)	0.000 **

Note: *p*-value for significance of independent *t*-test examining differences between boys and girls: * Significant effect *p* < 0.01; ** Significant effect *p* < 0.05.

**Table 3:** Results from linear regressions with fundamental movement skills as predictors of percentage body fat (adjusting for sex, maturity offset, ethnicity, deprivation).

Predictor	β	SE	95% CI	*p*	r^2^	sr_i_^2^
Total skill score	−0.27	0.08	−0.43 to −0.12	0.001	40.7%	5.2%
Locomotor skills	−0.88	0.14	−1.14 to −0.61	0.000	50.6%	15.0%
Object-control skills	0.26	0.12	0.02 to 0.51	0.036		2%

Notes. β = unstandardised regression coefficient, beta values reflect differences in percentage body fat for every 1 measured unit of each skill predictor variable; SE = standard error for β coefficient; 95% CI = 95% confidence intervals for regression coefficient; r^2^ = total variance explained by model (Model 1: total skill score, sex, maturity offset, ethnicity, deprivation; Model 2: Locomotor skill score, Object-control skill score, sex, maturity offset, ethnicity, deprivation score); sr_i_² = squared semi-partial correlation coefficient, unique variance explained by predictor.
